# Cytokine Profiling in Aqueous Humor Samples From Patients With Non-Infectious Uveitis Associated With Systemic Inflammatory Diseases

**DOI:** 10.3389/fimmu.2020.00358

**Published:** 2020-03-10

**Authors:** Martina Bonacini, Alessandra Soriano, Luca Cimino, Luca De Simone, Elena Bolletta, Fabrizio Gozzi, Francesco Muratore, Maria Nicastro, Lucia Belloni, Alessandro Zerbini, Luigi Fontana, Carlo Salvarani, Stefania Croci

**Affiliations:** ^1^Clinical Immunology, Allergy and Advanced Biotechnologies Unit, Azienda Unità Sanitaria Locale-IRCCS di Reggio Emilia, Reggio Emilia, Italy; ^2^Rheumatology Unit, Azienda Unità Sanitaria Locale-IRCCS di Reggio Emilia, Reggio Emilia, Italy; ^3^Ocular Immunology Unit, Azienda Unità Sanitaria Locale-IRCCS di Reggio Emilia, Reggio Emilia, Italy; ^4^Ophthalmology Unit, Azienda Unità Sanitaria Locale-IRCCS di Reggio Emilia, Reggio Emilia, Italy; ^5^Department of Surgery, Medicine, Dentistry and Morphological Sciences, With Interest in Transplants, Oncology and Regenerative Medicine, University of Modena and Reggio Emilia, Modena, Italy

**Keywords:** uveitis, cytokines, vasculitis, precision medicine, Behçet's disease, Vogt Koyanagi Harada disease, aqueous humor

## Abstract

Non-infectious uveitis are intraocular inflammatory conditions caused by dysregulated activation of the immune response without any detectable infectious agents. The aim of this study was to explore potential markers and therapeutic targets for two distinct types of non-infectious uveitis associated with Behçet's disease (BD) and Vogt Koyanagi Harada (VKH) disease. Concentrations of 27 cytokines were investigated in aqueous humor (AH) samples from patients with active uveitis vs. healthy controls (HC) (*n* = 10 patients with BD-associated uveitis; *n* = 10 patients with VKH-associated uveitis; *n* = 10 HC) using the Bio-Plex Pro^TM^ human cytokine group I panel. Additionally, leukocytes in AH samples were counted with hemocytometers and characterized by flow cytometry. Eleven cytokines were differentially expressed between patients with uveitis and HC with a median concentration greater than 10 pg/ml. IL-6, IP-10, G-CSF, and IFNγ showed higher concentrations in AH samples from both BD and VKH patients while IL-2, IL-8, IL-13, TNFα, eotaxin, IL-1ra showed statistically significant higher concentrations only in AH samples from BD patients. GM-CSF was the sole cytokine with an opposite profile showing decreased levels in AH samples from BD patients. IL-1ra and IL-6 were detected at higher frequencies in AH samples from BD and VKH patients compared with those from HC while IFNγ and TNFα were not detected in HC. The concentrations of IL-6, IL-8, IP-10, G-CSF, IFNγ, TNFα, eotaxin, IL-1ra positively correlated with the concentrations of leukocytes in AH, suggesting that such cytokines can be produced by immune cells and/or attract and/or promote proliferation and survival of immune cells in these types of uveitis. The correlation matrix of cytokine concentrations in AH samples revealed that IFNγ, TNFα, eotaxin, IL-6, G-CSF highly correlated each other. The ratios of cytokine concentrations between AH and plasma intra-individuals showed that IL-2, IL-6, IP-10, GM-CSF were increased intraocularly. In conclusion, AH sampling followed by multiplex analysis of cytokines should be fostered in non-infectious uveitis to identify cytokines dysregulated intraocularly in each individual laying the groundwork for precision medicine.

## Introduction

Uveitis are fearsome forms of intraocular inflammation, potentially causing visual impairment and blindness without a prompt diagnosis and therapy. The incidence is low, estimated at 17–52/100,000 people per year. Nevertheless, they account for ~10% of blindness worldwide (1). Uveitis can derive either from infectious agents (infectious uveitis) or from aberrant, deregulated activation of immune system (e.g., against self-proteins, environmental triggers, tissue damage) in absence of any detectable infectious agents (non-infectious uveitis). An etiological diagnosis of non-infectious uveitis is possible in most cases, from 47 to 75% depending on the studies and they are frequently associated with systemic immune-mediated diseases (2–4).

The management of infectious uveitis requires specific treatments against the infectious agents. On the contrary, the management of non-infectious uveitis represents a challenge for clinicians due to disease heterogeneity, still scarce knowledge on disease pathogenesis, and paucity of randomized controlled trials assessing the real efficacy of available drugs (5). Current guidelines for non-infectious uveitis are based on non-specific immunosuppression. Corticosteroids are recommended as first-line therapy, followed by immunosuppressive drugs in case of relapses, and with steroid-sparing purposes (6). Traditional immunosuppressive agents such as azathioprine, cyclosporine A, or mycophenolate mofetil are usually preferred in the case of posterior uveitis (7). Biological agents (e.g., those inhibiting the TNFα signaling and recombinant IFNα) are currently part of the therapeutic armamentarium (5, 8–10). In the incoming era of precision medicine, tailored treatment of uveitis remains to be defined (11).

Successful therapeutic strategies in non-infectious uveitis require to act on patients' immune responses. The identification of specific immune effectors associated to and/or responsible for the different types of uveitis is necessary in order to select the most promising among the available targeted-therapies or design new targeted-therapies on an individual basis. Immune profiling of patients with different non-infectious uveitis has indeed highlighted some immune effectors (e.g., cytokines, chemokines, immune cell subsets) in aqueous humor (AH) and peripheral blood possibly involved in uveitis pathogenesis and that may allow to differentiate the various types of uveitis (12, 13). These immune effectors could be exploited to implement precision medicine for treatment of non-infectious uveitis. However, further drivers of uveitis pathogenesis need to be discovered and data from single-center studies should be confirmed by other independent studies.

In this study we investigated the concentrations of 27 cytokines in AH from patients affected by two distinct types of non-infectious uveitis, associated with systemic inflammatory diseases: Behçet's disease (BD) and Vogt Koyanagi Harada (VKH) disease, both in active phase, to provide potential markers and therapeutic targets.

BD is a chronic systemic inflammatory vasculitis of unknown etiology characterized by recurrent episodes of oral aphthous ulcers, genital ulcers, non-granulomatous uveitis, retinal vasculitis, skin lesions, and other manifestations (14). VKH is a systemic autoimmune disease characterized by bilateral granulomatous panuveitis with or without auditory, neurological, and cutaneous manifestations, partly attributed to immune responses directed against one or more antigens expressed by melanocytes and retinal pigment epithelium (15). Both BD and VKH are rare diseases.

## Materials and Methods

### Cohorts of Patients and Healthy Controls

This is an exploratory, monocentric, independent study performed at the Azienda Unità Sanitaria Locale-IRCCS, Reggio Emilia, Italy, one of the national reference centers for BD. A cohort of 10 patients with BD-related uveitis and a cohort of 10 patients with VKH-related uveitis, both in active disease phase were enrolled. A cohort of 10 Caucasian subjects who underwent phacoemulsification intervention for cataract (*n* = 6) and cornea surgery (*n* = 4), not affected by any other concomitant inflammatory and/or infectious diseases nor with prior history of uveitis were recruited as healthy controls (HC). All BD patients satisfied the 1990 criteria for diagnosis of Behçet's disease (16). Diagnosis of VKH was based on the revised international diagnostic criteria (17). The median age for the BD cohort was 30 years (InterQuartile Range; IQR: 25–43) and gender distribution was: 8/10 males and 2/10 females. The median age for the VKH cohort was 47 years (IQR: 36–63) and gender distribution was 1/10 male and 9/10 females. The median age for the HC cohort was 64 years (IQR: 40–79) and gender distribution was: 6/10 males and 4/10 females.

Patients were examined with slit lamp biomicroscopy, indirect ophthalmoscopy, retinography, optical coherence tomography, fluorescein and indocyanine angiography. Patients with BD were considered as having active uveitis at ophthalmologic evaluation in case of non-granulomatous panuveitis with vitritis, or macular oedema, or occlusive retinal vasculitis, or retinal ischemia, or retinal and/or optic nerve neovascularization. Patients with VKH were considered as having active uveitis at ophthalmologic evaluation in case of granulomatous panuveitis with bilateral papillitis and exudative retinal neuroepithelium detachment with mild or absent vitreitis and choroidal granulomas. The features of uveitis for each patient are shown in Table 1. 5/10 BD patients and 2/10 VKH patients were receiving therapies at the moment of sample collection. The study was approved by the local ethics committee (Reggio Emilia, Italy, protocol number 2015/0024354) in compliance with the declaration of Helsinki and written informed consent was obtained from all patients and HC.

**Table 1:** Clinical characteristics of patients with BD and VKH-associated uveitis.

**Patients**	**Therapy**	**Anatomic location**	**Type of uveitis**	**Macular edema**	**Keratic precipitates**	**Vitreitis**	**Papillitis**	**Diffuse capillaritis**	**Exudative retinal detachment**	**Choroidal lesions**	**Vessel sheathing**
BD#1	No	Bilateral Panuveitis	Non-granulomatous	Yes	Yes	2	No	Yes	No	No	Yes
BD#2	Yes	Bilateral Panuveitis	Non-granulomatous	Yes	Yes	1	Yes	Yes	No	No	Yes
BD#3	Yes	Bilateral Panuveitis	Non-granulomatous	Yes	Yes	1	Yes	Yes	No	No	No
BD#4	Yes	Unilateral Panuveitis	Non-granulomatous	Yes	Yes	1	Yes	Yes	No	No	No
BD#5	Yes	Bilateral Panuveitis	Non-granulomatous	Yes	No	1	No	Yes	No	No	No
BD#6	No	Bilateral Panuveitis	Non-granulomatous	Yes	Yes	3	Yes	Yes	No	No	No
BD#7	No	Bilateral Panuveitis	Non-granulomatous	No	Yes	1	Yes	Yes	Yes	No	Yes
BD#8	Yes	Bilateral Panuveitis	Non-granulomatous	No	Yes	1	No	Yes	No	No	No
BD#9	No	Unilateral Panuveitis	Non-granulomatous	No	Yes	0,5	No	Yes	No	No	No
BD#10	No	Bilateral Panuveitis	Non-granulomatous	No	Yes	1	Yes	Yes	No	No	No
VKH#1	No	Bilateral Panuveitis	Granulomatous	No	No	No	Yes	No	Yes	Yes (5)	No
VKH#2	Yes	Bilateral Panuveitis	Granulomatous	No	No	No	Yes	No	Yes	Yes (5)	No
VKH#3	No	Bilateral Panuveitis	Granulomatous	No	No	No	Yes	No	Yes	Yes (5)	No
VKH#4	No	Bilateral Panuveitis	Granulomatous	No	No	No	Yes	No	Yes	Yes (5)	No
VKH#5	No	Bilateral Panuveitis	Granulomatous	Yes	No	No	Yes	No	Yes	No	No
VKH#6	No	Bilateral Panuveitis	Granulomatous	Yes	No	No	Yes	No	Yes	Yes (5)	No
VKH#7	No	Bilateral Panuveitis	Granulomatous	No	No	No	Yes	No	Yes	Yes (5)	No
VKH#8	No	Bilateral Panuveitis	Granulomatous	No	No	No	Yes	No	Yes	No	No
VKH#9	No	Bilateral Panuveitis	Granulomatous	No	No	No	Yes	No	Yes	Yes (5)	No
VKH#10	Yes	Bilateral Panuveitis	Granulomatous	No	Yes	No	Yes	No	Yes	Yes (5)	No

*Hypopyon was present only in BD#6. Retinitis was present only in BD#7 and BD#8. Snowbank was present only in BD#2. Snowballs were not present*.

### Biological Sample Collection

Samples of AH (100–200 μl) were obtained by anterior chamber paracentesis (18) conducted under surgical microscope. Ethylenediaminetetraacetic acid (EDTA) was added at 2 mM to prevent cell aggregation. Cell concentrations were determined by manual counting in Neubauer hemocytometers. AH samples were then centrifuged at 400 × g for 8 min. Cell pellets were analyzed by flow cytometry while cell-free supernatants were stored frozen at −80°C until cytokine profiling. Six milliliter of venous blood were collected from BD and VKH patients into EDTA coated tubes. Plasma samples were obtained by centrifugation at 1,300 × g for 20 min and stored at −80°C until use. Biosafety level 2 procedures were applied when working with patients' samples.

### Flow Cytometry

AH cells were suspended in 300 μL phosphate-buffered saline (PBS, Euroclone) + 1% heat inactivated fetal bovine serum (FBS, Thermo Fisher) and acquired with the FACSCanto II flow cytometer (BD Biosciences), equipped with two lasers for excitation at 488 and 633 nm. Data were analyzed with FACSDiva 8.0.1. software. Lymphocytes, monocytes, and granulocytes were identified according to the forward scatter (FSC) and side scatter (SSC) as shown in Figure S1.

### Cytokine Profiling

Concentrations of IL-1β, IL-1ra, IL-2, IL-4, IL-5, IL-6, IL-7, IL-8, IL-9, IL-10, IL-12p70, IL-13, IL-15, IL-17A, eotaxin, basic FGF, G-CSF, GM-CSF, IFNγ, IP-10, MCP-1, MIP-1α, MIP-1β, PDGF-BB, RANTES, TNFα, and VEGF were determined in AH samples using the Bio-Plex Pro™ human cytokine group I panel, 27-Plex (Bio-Rad) following the manufacturer's instruction. Cell-free AH samples were centrifuged at 10,000 × g for 10 min at 4°C and then diluted 4-fold in Bio-Plex sample diluent adding bovine serum albumin (BSA) at 0.5% as recommended for samples with low content of albumin. Plasma samples were centrifuged at 10,000 × g for 10 min at 4°C then were diluted 4-fold in Bio-Plex sample diluent. Eight serial dilutions of cytokine standards plus blanks (diluent) were included in each assay. Data were obtained with Bio-Plex MAGPIX^TM^ multiplex reader instrument and analyzed with Bio-Plex Manager^TM^ software. Standard curves were calculated with the five-parameter logistic equation regression method. Values extrapolated from the standard curves were considered not reliable and concentrations = 0.01 pg/ml were arbitrarily assigned to be able to draw graphs with logarithmic axes. The lower limits of cytokine detection are reported in Table S1.

### Clustering and Pathway Analysis

Clustering was performed using the ClustVis software (19) consisting of the following data pre-processing: (1) logarithmic transformation [ln (x+1)]; (2) row centering; (3) no scaling and applying the Euclidean complete distance for rows and columns. Pathway analysis was performed using REACTOME and PANTHER analysis software (reactome.org; pantherdb.org).

### Statistical Analysis

Statistical analysis was performed using GraphPad Prism 6 software. In order to compare two groups, non-parametric Mann-Whitney U-test was used for quantitative variables, while Fisher's exact test was used for qualitative variables. The Kruskal-Wallis test with Dunn's correction for multiple comparisons was used to compare cytokine concentrations in AH samples. Spearman's correlation was chosen to determine correlations between quantitative variables followed by the Bonferroni correction for multiple testing. *P* < 0.05 (two-tailed) were considered statistically significant.

## Results

### Cytokine Concentrations in AH Samples From BD and VKH Patients Compared to HC

To better understand ocular inflammatory *milieu* of patients with non-infectious uveitis, concentrations of 27 cytokines were determined in AH samples from patients with BD and VKH-associated uveitis in comparison with HC.

**Figure 1:**
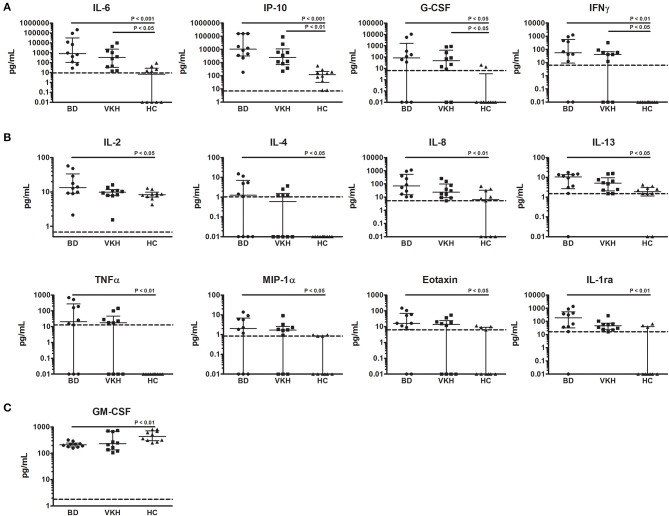
Cytokines differentially expressed in AH from patients with BD- and VKH-associated uveitis compared to HC. Dot plot visualization of cytokine concentrations (pg/ml) in AH from patients with BD (*n* = 10), VKH (*n* = 10), and HC (*n* = 10). **(A)** Cytokines expressed at higher levels both in BD and VKH. **(B)** Cytokines expressed at higher levels only in BD. **(C)** Cytokines expressed at lower levels in BD. Horizontal lines show the median ± IQR. Dotted lines indicate the lower limits of cytokine detection. Data were analyzed by the Kruskal-Wallis test with Dunn's correction for multiple comparisons.

IL-6, IP-10, G-CSF, and IFNγ showed higher concentrations in AH samples from both BD and VKH patients (Table 2 and Figure 1A). IL-6 and IP-10 were detected in all the patients' samples: IL-6 had 116-fold difference between BD and HC (*p* = 0.0004) and 48-fold difference between VKH and HC (*p* = 0.0153); IP-10 had 83 fold difference between BD and HC (*p* = 0.0001) and 20-fold difference between VKH and HC (*p* = 0.0063). Moreover, IL-2, IL-4, IL-8, IL-13, TNFα, MIP-1α, eotaxin, IL-1ra showed statistically significant higher concentrations in AH samples from BD patients compared to HC (Table 2 and Figure 1B). GM-CSF was the unique cytokine with an opposite profile, revealing lower concentrations in AH samples from BD patients compared to HC (Table 2). AH samples from VKH patients showed a bimodal distribution regarding GM-CSF and 6/10 patients had concentrations lower than HC (Figure 1C).

**Table 2:** Concentrations of cytokines in AH from BD patients, VKH patients and HC.

	**Concentration (pg/mL)**			***P*****-value**		
	**BD** ***n* = 10**	**VKH** ***n* = 10**	**HC** ***n* = 10**	**BD vs. HC**	**VKH vs. HC**	**BD vs. VKH**
IL-1β	0.01 (0.01–12.63)	0.01 (0.01–0.85)	n.d.	n.s.	n.s.	n.s.
IL-1ra	185.80 (31.64–534)	46.99 (24.83–74.54)	0.01 (0.01–39.96)	0.0053	n.s.	n.s.
IL-2	13.32 (9.16–33.60)	9.88 (7.96–11.69)	8.32 (7.13–9.83)	0.0384	n.s.	n.s.
IL-4	1.24 (0.01–6.97)	0.60 (0.01–1.53)	n.d.	0.0118	n.s.	n.s.
IL-5	0.01 (0.01–8.42)	0.01 (0.01–1.18)	n.d.	n.s.	n.s.	n.s.
IL-6	812.2 (107.5–32478)	333.70 (33.13–2303)	6.79 (0.01–27.66)	0.0004	0.0153	n.s.
IL-7	14.43 (0.01–28.99)	5.00 (0.01–15.46)	0.01 (0.01–4.67)	n.s.	n.s.	n.s.
IL-8	69.46 (15.48–532.3)	24.15 (9.18–97.47)	6.53 (0.01–33.14)	0.0088	n.s.	n.s.
IL-9	8.36 (2.85–10.65)	2.85 (0.01–8.50)	1.23 (0.01–4.92)	n.s.	n.s.	n.s.
IL-10	14.41 (10.51–23.84)	10.43 (8.75–14.57)	10.39 (8.71–12.69)	n.s.	n.s.	n.s.
IL-12 (p70)	27.36 (13.82–54.45)	15.40 (8.80–27.85)	18.15 (0.01–32.33)	n.s.	n.s.	n.s.
IL-13	10.43 (2.66–14.04)	5.06 (2.08–9.46)	1.87 (1.11–3.05)	0.0263	n.s.	n.s.
IL-15	0.01 (0.01–108.9)	12.12 (0.01–70.30)	47.45 (0.01–65.57)	n.s.	n.s.	n.s.
IL-17A	17.00 (13.16–69.00)	20.83 (11.57–28.16)	22.03 (13.45–27.81)	n.s.	n.s.	n.s.
Eotaxin	16.27 (6.63–70.58)	13.83 (0.01–24.22)	0.01 (0.01–9.13)	0.0260	n.s.	n.s.
Basic FGF	85.24 (57.83–127.80)	65.29 (34.83–88.79)	84.37 (70.57–106.40)	n.s.	n.s.	n.s.
G-CSF	85.19 (0.01–1638)	46.17 (6.48–393.40)	0.01 (0.01–3.31)	0.0176	0.0305	n.s.
GM-CSF	209.30 (175.40–244.10)	230.80 (133.30–683.40)	438.40 (299.10–709.50)	0.0072	n.s.	n.s.
IFNγ	54.82 (9.26–574.70)	41.46 (0.01–66.48)	n.d.	0.0011	0.0321	n.s.
IP-10	10,311 (3,172–155,581)	2,400 (724.3–10,990)	123.20 (31.59–214.80)	0.0001	0.0063	n.s.
MCP-1	397.6 (128.9–2,306)	323.6 (167.60–626.40)	235.20 (114.40–420.30)	n.s.	n.s.	n.s.
MIP-1α	2.04 (0.01–6.81)	1.69 (0.01–2.56)	0.01 (0.01–0.87)	0.0407	n.s.	n.s.
MIP-1β	41.63 (9.13–124.90)	19.06 (14.5–29.78)	35.23 (8.16–61.61)	n.s.	n.s.	n.s.
PDGF-BB	8.54 (0.01–26.34)	2.24 (0.01–10.22)	0.01 (0.01–2.81)	n.s.	n.s.	n.s.
RANTES	7.63 (0.01–48.72)	0.01 (0.01–0.01)	0.01 (0.01–17.24)	n.s.	n.s.	n.s.
TNFα	20.48 (0.01–268.80)	17.74 (0.01–46.59)	n.d.	0.0055	n.s.	n.s.
VEGF	58.44 (34.66–116.70)	49.90 (26.39–85.33)	65.62 (28.95–108.60)	n.s.	n.s.	n.s.

*n.d., non detectable; n.s., not significant. Cytokines with concentrations below the lower limits of detection were considered as non-detected and concentrations = 0.01 pg/ml were arbitrarily assigned. Data were analyzed with the Kruskal-Wallis test with Dunn's correction for multiple comparisons. Median concentrations with interquartile ranges (in brackets) are shown*.

IL-1β, IL-5, IL-7, IL-9, IL-10, IL-12p70, IL-15, IL-17, MCP-1, MIP-1β, PDGF-BB, RANTES, VEGF, basic FGF showed comparable levels among groups (Table 2 and Figure S2). Moreover, no differences in AH cytokine levels were found between BD and VKH patients (Table 2).

It must be underlined that some patients were receiving immunosuppressive therapies at the moment of AH collection despite the presence of active uveitis. To investigate if therapies affected cytokine concentrations, we compared AH samples from patients with and without therapies. This analysis was feasible only in the cohort of BD patients which included 5 patients under therapies and 5 patients naïve from therapies (Table 1). MCP-1, IL-7, IL-8, G-CSF, MIP-1α, MIP-1α, TNFα, IFNγ showed higher concentrations and GM-CSF showed lower concentrations in AH from patients without therapies (Figure S3) but the statistical significances were not maintained after correction for multiple testing.

To investigate if some cytokines were detected more frequently in AH from patients than from HC, the percentages of AH samples with detectable levels of cytokines were compared among the cohorts. IL-1ra, IL-4, IL-6, IFNγ, and TNFα were detected at higher frequencies in AH samples from BD and VKH patients compared with those from HC (Table 3). In particular, IL-4, IFNγ and TNFα were not detected in any of the samples from HC.

**Table 3:** Presence of cytokines in AH from BD patients, VKH patients and HC.

	**Positive sample (fraction)**			**Fisher test (*****p*****-value)**		
	**BD** ***n* = 10**	**VKH** ***n* = 10**	**HC** ***n* = 10**	**BD vs. HC**	**VKH vs. HC**	**BD vs. VKH**
IL-1β	4/10	2/10	0/10	n.s.	n.s.	n.s.
IL-1ra	9/10	10/10	3/10	0.0198	0.0031	n.s.
IL-2	10/10	10/10	10/10	n.s.	n.s.	n.s.
IL-4	6/10	5/10	0/10	0.0108	0.0325	n.s.
IL-5	4/10	2/10	0/10	n.s.	n.s.	n.s.
IL-6	10/10	10/10	5/10	0.0325	0.0325	n.a.
IL-7	7/10	6/10	3/10	n.s.	n.s.	n.s.
IL-8	10/10	10/10	6/10	n.s.	n.s.	n.a.
IL-9	9/10	7/10	5/10	n.s.	n.s.	n.s.
IL-10	9/10	10/10	9/10	n.s.	n.s.	n.s.
IL-12 (p70)	8/10	9/10	7/10	n.s.	n.s.	n.s.
IL-13	9/10	10/10	8/10	n.s.	n.s.	n.s.
IL-15	4/10	5/10	7/10	n.s.	n.s.	n.s.
IL-17A	10/10	9/10	10/10	n.a.	n.s.	n.s.
Eotaxin	8/10	6/10	4/10	n.s.	n.s.	n.s.
Basic FGF	10/10	10/10	10/10	n.a.	n.a.	n.a.
G-CSF	7/10	8/10	2/10	n.s.	0.0230	n.s.
GM-CSF	10/10	10/10	10/10	n.a.	n.a.	n.a.
IFNγ	8/10	7/10	0/10	0.0007	0.0031	n.s.
IP-10	10/10	10/10	10/10	n.a.	n.a.	n.a.
MCP-1	10/10	10/10	10/10	n.a.	n.a.	n.a.
MIP-1α	7/10	7/10	4/10	n.s.	n.s.	n.s.
MIP-1β	10/10	10/10	10/10	n.a.	n.a.	n.a.
PDGF-BB	6/10	5/10	3/10	n.s.	n.s.	n.s.
RANTES	5/10	1/10	4/10	n.s.	n.s.	n.s.
TNFα	7/10	6/10	0/10	0.0031	0.0108	n.s.
VEGF	9/10	9/10	9/10	n.s.	n.s.	n.s.

*n.a., not applicable; n.s., not significant. The numbers of samples in which cytokine concentrations were equal to or higher than the respective lower limits of detection are shown*.

No differences in terms of frequencies of cytokine detection in AH samples were found between BD and VKH patients (Table 3).

Subsequent analyses were focused on the 11 cytokines which resulted differentially expressed between patients with uveitis and HC and, to have a higher degree of confidence, which showed median concentrations greater than 10 pg/ml. To identify cytokines with similar profiles, we calculated the correlation matrix of cytokine concentrations in the AH samples. IFNγ, TNFα, eotaxin, IL-6, G-CSF highly correlated each other (*p* < 10^−6^) (Table 4).

**Table 4:** Correlation matrix of cytokine concentrations in AH samples from patients with BD- and VKH-associated uveitis.

	**IL-1ra**	**IL-2**	**IL-6**	**IL-8**	**IL-13**	**Eotaxin**	**G-CSF**	**GM-CSF**	**IFNγ**	**IP-10**
IL-2	0.424308									
IL-6	0.001086	0.568329								
IL-8	0.002796	1.000000	0.000662							
IL-13	1.000000	0.212626	0.896414	1.000000						
Eotaxin	0.008220	0.663621	0.000000	0.005417	0.252160					
G-CSF	0.000338	0.445325	0.000000	0.015374	0.314126	0.000000				
GM-CSF	1.000000	1.000000	0.311010	0.176605	0.019136	0.052681	0.192300			
IFNγ	0.000008	0.184949	0.000000	0.000399	0.252101	0.000000	0.000000	0.122415		
IP-10	0.015567	1.000000	0.079025	0.000099	1.000000	0.020413	0.184789	0.097607	0.006462	
TNFα	0.001325	0.348987	0.000000	0.001020	0.258832	0.000000	0.000000	0.093605	0.000000	0.014286

*Spearman's correlation was calculated for every pair of data set. P-values are shown after the Bonferroni correction*.

To investigate which cytokines had higher levels in the ocular environment, cytokine concentrations in AH were compared to those in plasma intra-individuals. GM-CSF and IL-6 showed higher levels in AH from all the patients, IL-2 from 19/20 patients and IP-10 from 16/20 patients. Specifically, IL-2 was detected only in AH samples from 18 patients and IL-6 was detected only in AH samples from 12 patients. On the contrary, eotaxin showed lower levels in AH from 16/20 patients and was detected only in plasma from 6 patients. The other cytokines showed heterogeneous patterns (Table 5). Looking at the AH over plasma cytokine profiles across patients, BD patient#9 displayed higher concentrations in AH of all the cytokines while BD patient#2, BD patient#7 and VKH patient#7 showed higher concentrations in AH of 10/11 cytokines with the exception of eotaxin (Table 5).

**Table 5:** Ratios between cytokine concentrations in AH and plasma for each patient.

	**IL-6**	**IP-10**	**G-CSF**	**IFNγ**	**IL-2**	**IL-8**	**IL-13**	**TNFα**	**Eotaxin**	**IL-1ra**	**GM-CSF**
BD#1	Only AH	14.6	5.8	2.4	Only AH	10.7	1.3	1.7	−2.6	1.5	6.7
BD#2	Only AH	794.7	34.9	Only AH	Only AH	Only AH	1.8	Only AH	−1.5	18.3	4.3
BD#3	Only AH	11.9	Non detected	Only AH	Only AH	Only AH	−2.9	Non detected	Only plasma	Only AH	4.3
BD#4	Only AH	11.1	1.6	−1.8	Only AH	2.4	−3.3	−2.2	−2.7	−1.3	5.9
BD#5	7.7	−3.2	Only plasma	Only plasma	Only AH	−1.1	−3.4	Only plasma	Only plasma	−9.0	7.0
BD#6	7,360.6	151.0	92.1	2.2	3.6	27.1	−1.1	4.3	1.2	3.1	2.4
BD#7	Only AH	184.1	Only AH	Only AH	Only AH	Only AH	1.9	Only AH	1.3	Only AH	3.2
BD#8	1.4	3.8	Only plasma	Only plasma	−1.5	−2.5	−7.8	Only plasma	−27.0	Only plasma	6.6
BD#9	Only AH	5.0	713.9	Only AH	Only AH	Only AH	3.9	Only AH	2.8	20.3	Only AH
BD#10	Only AH	43.8	Only AH	Only AH	Only AH	Only AH	Non detected	Only AH	−3.8	Only AH	Only AH
VKH#1	Only AH	13.0	14.2	1.0	Only AH	5.7	−7.6	1.1	−3.2	−1.2	4.4
VKH#2	3.9	10.6	Only plasma	Only plasma	Only AH	1.2	−5.1	Only plasma	Only plasma	−29.3	46.2
VKH#3	Only AH	2.5	−1.9	−11.2	Only AH	1.1	1.1	Only plasma	Only plasma	−1.2	5.9
VKH#4	Only AH	39.7	2.0	1.0	Only AH	25.0	1.8	Only AH	−6.2	−2.1	5.9
VKH#5	Only AH	5.0	Only AH	Only AH	Only AH	Only AH	Only AH	Only AH	−2.2	Only AH	3.3
VKH#6	34.3	−1.1	2.3	−1.9	Only AH	2.3	3.8	−1.2	−4.3	−3.7	Only AH
VKH#7	Only AH	59.6	Only AH	Only AH	Only AH	Only AH	Only AH	Only AH	−1.1	Only AH	Only AH
VKH#8	612.9	−2.0	8.9	−2.3	Only AH	1.2	−7.0	−1.2	−2.4	−2.4	16.4
VKH#9	1.7	8.8	Only plasma	Only plasma	Only AH	−3.1	−3.4	Only plasma	Only plasma	−3.5	Only AH
VKH#10	3.0	−1.3	−8.7	Only plasma	Only AH	−2.6	−6.0	Only plasma	Only plasma	−4.1	33.3

*Ratios between cytokine concentrations in AH and plasma are reported. In case the ratios were below 1 the following formula was applied:-−1/ratio. Therefore, negative values indicate a higher expression of the cytokines in plasma, positive values indicate a higher expression in AH. “Only AH,” cytokines detected only in AH samples. “Only plasma,” cytokines detected only in plasma samples. “Non detected,” cytokines detected neither in AH nor in plasma samples*.

### Correlation Between Cytokine Concentrations and Loads of Inflammatory Cells in AH Samples

**Figure 2:**
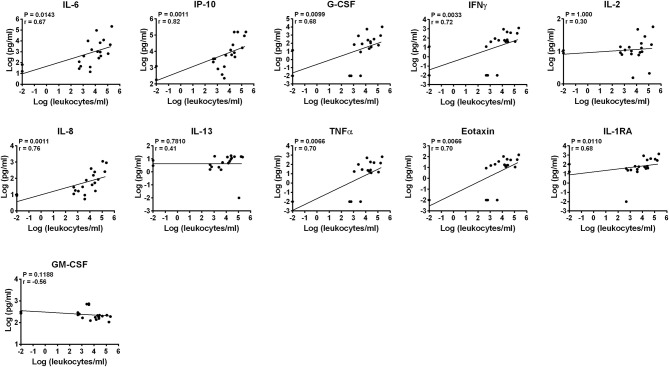
Correlation of cytokine concentrations with the load of leukocytes in AH. Correlations between log-scale cytokine concentrations and leukocyte concentrations in AH samples are depicted (*n* = 20). Spearman's correlation was determined. *P*-values after the Bonferroni correction for multiple testing are shown.

Concentrations of leukocytes in AH samples were assessed by manual counting with hemocytometers while concentrations of lymphocytes, monocytes and granulocytes (neutrophils) were semi-quantitatively estimated applying their percentages obtained by flow cytometry (Figure S1). Samples were heterogeneous in terms of concentrations of infiltrating leukocytes and two samples did not show any cells (Table 6). The median concentration of leukocytes in AH from BD patients did not differ from that of VKH patients (24,700 cells/ml, IQR: 470–63,375 vs. 3,750 cells/ml, IQR: 1,004–16,050). Concentrations of IL-6, IL-8, IP-10, G-CSF, IFNγ, TNFα, eotaxin, IL-1ra positively correlated with concentrations of leukocytes. Among them, IP-10 and IL-8 showed the best correlations (*p* = 0.0011, *r* = 0.82 and *r* = 0.76). Instead IL-2, IL-13, and GM-CSF levels did not correlate with leukocyte concentrations (Figure 2).

**Table 6:** Characteristics of the immune cells present in the AH samples.

**Patients**	**Leukocytes/ml**	**Lymphocytes (%)**	**Monocytes (%)**	**Granulocytes (%)**	**Lymphocytes/ml§**	**Monocytes/ml#**	Granulocytes/ml^**^
BD#1	25,000	84	13	2	+++	++	++
BD#2	47,500	62	33	4	+++	+++	+++
BD#3	470	64	25	11	+	+	+
BD#4	28,100	80	18	1	+++	+++	++
BD#5	0	0	0	0	0	0	0
BD#6	220,000	24	11	65	+++	+++	+++
BD#7	24,400	46	16	38	++	+++	+++
BD#8	470	70	5	24	+	+	+
BD#9	12,500	50	8	42	++	++	+++
BD#10	111,000	52	17	31	+++	+++	+++
VKH#1	15,000	82	15	3	++	++	++
VKH#2	630	72	11	17	+	+	+
VKH#3	0	0	0	0	0	0	0
VKH#4	19,200	80	18	2	++	++	++
VKH#5	5,000	68	26	6	++	++	+
VKH#6	1,129	34	39	27	+	++	+
VKH#7	166,250	93	6	1	+++	+++	+++
VKH#8	2,500	38	14	48	++	++	++
VKH#9	3,750	85	7	9	++	+	++
VKH#10	3,750	57	27	17	++	++	++

*Leukocytes/ml were determined by counting with Neubauer hemocytometers. Percentages of lymphocytes, monocytes and granulocytes were determined by flow cytometry setting gates based on FSC and SSC. Such percentages were applied to the leukocytes/ml to estimate lymphocytes/ml, monocytes/ml and granulocytes/ml. Concentrations of such immune cell subsets were divided in three classes: ≤33% percentile; 33–67% percentile; ≥67% percentile to semi-quantitatively assess the immune cell loads*.

§ *: +, ≤900 lymphocytes/ml; ++, 900–16,000 lymphocytes/ml; +++, ≥16,000 lymphocytes/ml*.

# *: +, ≤350 monocytes/ml; ++, 350–3,500 monocytes/ml; +++, ≥3,500 monocytes/ml*.

** *: +, ≤300 granulocytes/ml; ++, 300–1,200 granulocytes/ml; +++, ≥1,200 granulocytes/ml*.

*Samples of AH from HC did not contain any leukocytes*.

Correlation analyses between cytokine concentrations and leukocyte subsets (Table 6) showed that IL-6, IL-8, IP-10, G-CSF, IFNγ, TNFα, eotaxin, IL-1ra concentrations positively correlated also with the semi-quantitative degrees of lymphocytes/ml, monocytes/ml and neutrophils/ml in AH. Higher correlations (lower *p*-values and higher *r* values) were found with the degrees of monocytes and neutrophils compared to those of lymphocytes (Figures S4–S6). In particular, the best correlations (*p* = 0.0011, *r* = 0.75) were shown by IL-8 and IFNγ with the semi-quantitative densities of neutrophils/ml.

Finally, cytokine concentrations did not correlate with the percentages of lymphocytes, monocytes and neutrophils in AH (data not shown).

### Unsupervised Cluster Analysis and Pathway Analysis

**Figure 3:**
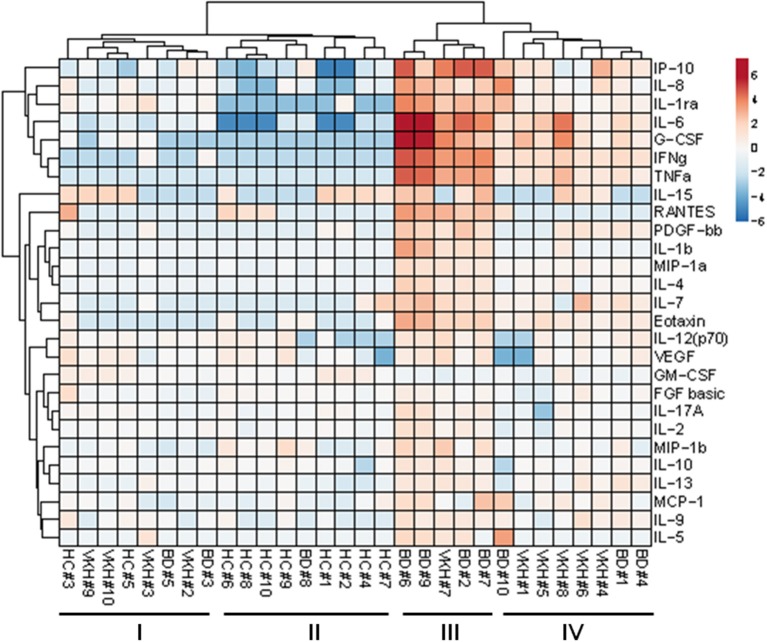
Unsupervised clustering. Clustering was performed with ClustVis software.

To explore possible clustering of the subjects based on the 27 cytokine profiles in AH samples, unsupervised cluster analysis was performed (Figure 3). Subjects were clustered in 2 major groups: the first one composed of all HC plus 7 BD and VKH patients; the second one composed only of BD and VKH patients (*n* = 13). Within such two groups other two clusters appeared. In particular, cluster II contained 9/10 HCs and BD#8 whose AH sample derived from a blinded eye with few leukocytes/ml (Table 6). 5/7 patients in cluster I-II vs. 2/13 patients in cluster III-IV were receiving therapies at the moment of AH sampling (*p* = 0.0223, Fisher's exact test). Patients in cluster I-II had lower leukocytes/ml than patients in cluster III-IV (470 cells/ml, IQR: 0–3,750 vs. 24,400 cells/ml, IQR: 8,750–79,250; *p* = 0.0011, Mann-Whitney U-test). Finally, cluster III included 5/6 patients with higher neutrophils/ml (score = +++, Table 6). We were unable to find other common clinical characteristics (Table 1) in the clustered patients.

Cytokines were clustered in two major groups: the first one included cytokines with the highest fold difference in concentrations between patients with uveitis and controls: IL-6, IL-8, IFNγ, TNFα, IP-10, G-CSF, IL-1ra (Figure 3).

To appreciate cytokine profiles in the clinical diagnostic clusters, a heatmap displaying cytokine concentrations in AH from each subject classified as BD, VKH and HC was drawn (Figure S7).

Loading the list of the 11 dysregulated cytokines in REACTOME and PANTHER software produced respectively the “interleukin-10 signaling pathway” as top over-represented pathway and “granulocyte and leukocyte chemotaxis” as biological process.

## Discussion

The present study proposes multiplex analysis of cytokine concentrations in AH from patients with non-infectious uveitis vs. HC, as a tool to identify cytokines deregulated intraocularly in each individual, in order to gain insight in uveitis pathogenesis and explore potential targets for the development of tailored treatments.

Concentrations of the deregulated cytokines: IL-6, IL-8, IP-10, G-CSF, IFNγ, TNFα, eotaxin, IL-1ra positively correlated with the levels of inflammatory cells in AH. This was expected supposing that cytokines in AH from patients with uveitis are produced by leukocytes and/or attract leukocytes and/or sustain leukocyte survival and proliferation. Moreover it is in line with findings by other authors regarding positive correlations between cytokine levels in AH and disease activity in patients with BD- and VKH-associated uveitis, graded according to the criteria of the Standardization of Uveitis Nomenclature Working Group (20–23). Such cytokines can be produced by and/or attract monocytes/macrophages and neutrophils. This is consistent with the stronger correlations found between cytokine levels and the degrees of monocytes and granulocytes in AH, which points to a possible role of the innate immune system in BD- and VHK-associated uveitis.

Concentrations of IL-2 and IL-13 did not correlate with the loads of inflammatory cells. That was unexpected because CD4+ and CD8+ T cells, NK cells, and dendritic cells are the major sources of IL-2, while CD4+ T cells, NKT cells, mast cells, basophils and eosinophils are the major sources of IL-13. However, these findings could be explained by production of IL-2 and IL-13 also by ocular cells, supported by the fact that IL-2 and IL-13 were detected also in AH from HC that normally did not contain leukocytes. Thus, we might speculate that IL-2 and IL-13 play a role not only in inflammation but also in ocular homeostasis.

Another cytokine which could be produced also by ocular cells is GM-CSF, being highly detected in AH from HC. The decreased levels of GM-CSF in AH from patients with non-infectious uveitis may suggest eye-protective, anti-inflammatory effects of such a cytokine. GM-CSF can be secreted by both immune cells (e.g., macrophages, T lymphocytes, NK cells) and stromal cells (e.g., endothelial cells and fibroblasts). It is generally thought as a pro-inflammatory cytokine playing as a growth and differentiation factor for granulocytes and macrophages. However, it can also promote immunological tolerance (24, 25) and induce the expression of the immune checkpoint molecule PD-L1 dampening immune responses (26). We may suppose that GM-CSF is involved in the maintenance of ocular *immune privilege*, a phenomenon restraining local immune and inflammatory responses in order to preserve vision through physical barriers (i.e., blood–ocular barrier), soluble and surface-bound molecules and modulation of systemic immune responses (27).

The detection of a cytokine does not imply that it plays a role in disease pathogenesis. However the identification of which cytokines are deregulated in each patients can provide a rationale to select targeted therapies, in particular the exclusion of the drugs whose targets are not deregulated or detected in individual patients.

The specific presence of IFNγ and TNFα in AH samples only from patients with uveitis renders these cytokines promising targets in a therapeutic perspective. Indeed, biological drugs anti-TNFα have been proven to be effective in non-infectious uveitis and have been approved by FDA and EMEA for therapy (6, 10, 28–32). Inhibitors of IFNγ (e.g., AMG811; Fontolizumab, Emapalumab) are being evaluated in clinical trials on immune mediated diseases. Our results suggest that they might be also evaluated for treatment of patients with non-infectious uveitis.

To be noted, administration of recombinant IFNα which belongs to the type I interferons, has been proved to be effective in patients with BD-related uveitis refractory to conventional immunosuppressive treatment (33, 34). IFNα has anti-viral, anti-proliferative, anti-angiogenic and anti-tumor activities and can modulate the immune system. Although there are not evidences that BD results from direct infection by viruses or bacteria, many data suggest an important role for infective agents as triggers of the immune-responses observed in BD (35). Administration of IFNα seems to (1) shape polarization of CD4+ lymphocytes toward Th2; (2) decrease Th17 lymphocytes, γδ T lymphocytes and NK cells; (3) reduce the expression of Toll-like receptors on CD4+ T lymphocytes and monocytes; (4) inhibit neovascularization; (5) enhance functions of blood-ocular barrier (36–39).

The great differences in the concentrations of IP-10, IL-6, and G-CSF between AH from patients with uveitis and HC (at least 20-fold), in the majority of the patients, make them top candidate therapeutic targets as well. Therefore it could be interesting to test whether biological drugs such as anti-IP10 (e.g., eldelumab BMS-936557, MDX-1100), anti-IL-6 (e.g., tocilizumab, sarilumab, siltuximab, sirukumab, olokizumab, clazakizumab), anti-G-CSF (e.g., CSL324) might be beneficial in non-infectious uveitis. In support of such hypothesis, the anti-IL-6 receptor monoclonal antibody tocilizumab has already shown some efficacy in patients with non-infectious uveitis (40–42).

To the best of our knowledge, this is the first report about an increased production of IL-1ra in BD-associated uveitis. IL-1ra is a natural endogenous inhibitor of the pro-inflammatory effect of IL-1β through the binding IL-1 receptor. IL-1ra is mainly produced by monocytes, neutrophils, epithelial cells, and keratinocytes. An increased production of IL-1ra has been documented also in AH from patients with HLA-B27-associated anterior uveitis (43). The increased production of an inhibitor of inflammation could represent a feedback loop to dampen the inflammatory responses. The biological drug Anakinra is a recombinant, slightly modified version of IL-1ra and its efficacy has been reported in the management of BD-related uveitis (44). Whether Anakinra efficacy might depend on the baseline levels of IL-1ra in AH is unknown. Since AH cytokine levels were heterogeneous among patients, clinical trials are strongly needed to verify the hypothesis of beneficial effects of different class of biological therapies in patients with non-infectious uveitis based on the levels of the respective targets in AH.

To identify which cytokines can be consistently dysregulated in AH from patients with uveitis associated with BD and VKH, we compared our data with literature data (Table 7). IL-1ra, G-CSF, IL-9, PDGF, and basic FGF were investigated for the first time in the present work, whereas the other cytokines have been previously investigated by other groups. Increased levels of IL-6, IL-8, IFNγ, IP-10 are confirmed respectively by four, nine, six and three independent studies (20–22, 45, 46, 48–56). Three studies confirm higher levels of TNFα in AH (20, 47, 48), while three studies are discordant with our results (45, 46, 49).

**Table 7:** Literature data about the expression of the investigated cytokines in AH from patients with BD- and VKH- associated uveitis compared to HC.

**Cytokine**	**Our data**	**Literature data**	**References**
IL-1β	=^*^	nd/↑/=/=	(22, 45–47)
IL-1ra	↑	no data	
IL-2	↑	nd/nd/=/=/↑	(45, 46, 48–50)
IL-4	↑^**^	nd/nd/↓/=/=	(45, 46, 48–50)
IL-5	=^*^	nd/nd/=	(45, 46, 49)
IL-6	↑	↑/↑/↑/↑	(22, 45, 46, 51)
IL-7	=	=	(46)
IL-8/CXCL8	↑	↑/↑/↑/↑/↑/↑/↑/↑/↑	(21, 45, 46, 51–56)
IL-9	=^**^	no data	
IL-10	=	↓/=/=/=/nd/nd/↑/↑	(20, 45–50, 52)
IL-12p70	=	=/=/=/=	(23, 45, 46, 48)
IL-13	↑	=	(46)
IL-15	=^*^	↑/↑/=	(20, 46, 48)
IL-17	=	↑	(20)
Eotaxin/CCL11	↑	=/↑	(46, 57)
FGF basic	=	no data	
G-CSF	↑	no data	
GM-CSF	↓	↑/=	(23, 46)
IFNγ	↑	↑/↑/↑/↑/↑/↑	(20, 45, 46, 48–50)
IP-10/CXCL10	↑	↑/↑/↑	(21, 54, 55)
TNFα	↑	↑/↑/↑/nd/nd/=	(20, 45–49)
MCP-1/CCL2	=	=/↑/↑/↑	(46, 51, 53, 57)
MIP-1α/CCL3	↑^**^	=	(53)
MIP-1β/CCL4	=	=	(53)
PDGF	=^**^	no data	
RANTES/CCL5	=^*^	=/=/=	(46, 53, 55)
VEGF	=	=/↑/↑	(51, 52, 56)

*Only studies including BD-associated uveitis and VKH-associated uveitis compared with HC are reported. ↑, increased production; ↓, decreased production; nd, not detected; =, unchanged*.

* *cytokines with median concentrations lower than the limits of detection*.

** *cytokines with median concentrations lower than 10 pg/ml*.

The profile of the other cytokines is heterogeneous in the literature and not consistently in line with our results (Table 7). Differences in the results can derive from technical aspects (e.g., types of assays used to quantify the cytokines; processing of AH samples: cell-free AH samples vs. whole AH samples) and from differences in the clinical characteristics of the cohorts of patients (e.g., therapies at the moment of sample collection; degree of uveitis). Efforts are needed (1) to standardize the protocols inter-laboratories using common cytokine standards to have data which can be compared among laboratories; (2) to define the range of cytokine concentrations in AH from HC to have references of non-inflammatory conditions; (3) to promote multicentric studies.

Merging our data with literature data (Table 7) pointed out that IL-8 could be an additional promising target for therapies in non-infectious uveitis. IL-8 can induce chemotaxis in neutrophils and other granulocytes, stimulate phagocytosis and promote angiogenesis. Inhibitors of IL-8 (e.g., BMS-986253), currently in development for treatment of some solid tumors, might be also tested in non-infectious uveitis.

Limits of the present study are (1) the small number of patients (although that is in line with the other studies on cytokine profiling in AH from patients with non-infectious uveitis); (2) the heterogeneity in terms of treatment schedule and degree of ocular inflammation at the moment of AH sample collection.

On the other hand, the strengths of the study mainly consist in the accurate clinical evaluation performed by expert rheumatologists and immune-ophthalmologists for defining BD activity and uveitis activity respectively, apart from simultaneous profiling of several cytokines and the characterization of the immune cells in AH by manual counting and flow cytometry, which has been rarely performed in other studies.

To resume, AH sampling followed by cytokine profiling allows identifying potential therapeutic targets for non-infectious uveitis and could help stratify patients for tailored treatments. IP-10, IFNγ, IL-6, G-CSF, TNFα IL-8, IL-1ra, and GM-CSF emerged as the most promising cytokines to be further investigated for treatment of BD- and VKH-associated uveitis.

## Data Availability Statement

The datasets generated for this study are available on request to the corresponding author.

## Ethics Statement

The studies involving human participants were reviewed and approved by the local ethics committee (Reggio Emilia, Italy, protocol number 402 2015/0024354). The patients/participants provided their written informed consent to participate in this study.

## Author Contributions

AS, LC, and SC designed the experimental protocol. AS, LC, LD, EB, FG, FM, LF, and CS recruited patients. MB, SC, and MN performed the experiments. MB, LB, AZ, CS, and SC interpreted the data. SC wrote the manuscript. All authors drafted the manuscript or revised it critically for important intellectual content and approved the final version to be published.

### Conflict of Interest

The authors declare that the research was conducted in the absence of any commercial or financial relationships that could be construed as a potential conflict of interest.
