# Supplementary material for: Cytokine Profiling in Aqueous Humor Samples From Patients With Non-Infectious Uveitis Associated With Systemic Inflammatory Diseases
Source: Front Immunol. 2020 Mar 10;11:358. doi: 10.3389/fimmu.2020.00358 (PMC7077343; doi:10.3389/fimmu.2020.00358)
Supplement: Supplementary file 1 [file Data_Sheet_1.pdf]

Supplementary Material

Table S1. Lower limits of detection (LLOD) of the investigated cytokines. LLOD corresponded to the concentrations of the most diluted standards whose signals (median fluorescence intensities) were reliably differentiated from blank values. Cytokine concentrations in AH samples below the LLOD were considered not reliable and values = 0.01 were arbitrary assigned.

	Concentration (pg/mL)
**IL-1β**	2.00
**IL-1ra**	16.64
**IL-2**	0.68
**IL-4**	1.04
**IL-5**	4.72
**IL-6**	9.20
**IL-7**	3.64
**IL-8**	5.36
**IL-9**	2.44
**IL-10**	6.68
**IL-12 (p70)**	7.48
**IL-13**	1.52
**IL-15**	22.80
**IL-17A**	4.40
**Eotaxin**	6.44
**Basic FGF**	8.12
**G-CSF**	6.28
**GM-CSF**	1.80
**IFNγ**	6.28
**IP-10**	6.96
**MCP-1**	4.96
**MIP-1α**	0.84
**MIP-1β**	3.02
**PDGF-BB**	1.92
**RANTES**	11.44
**TNFα**	12.88
**VEGF**	6.52

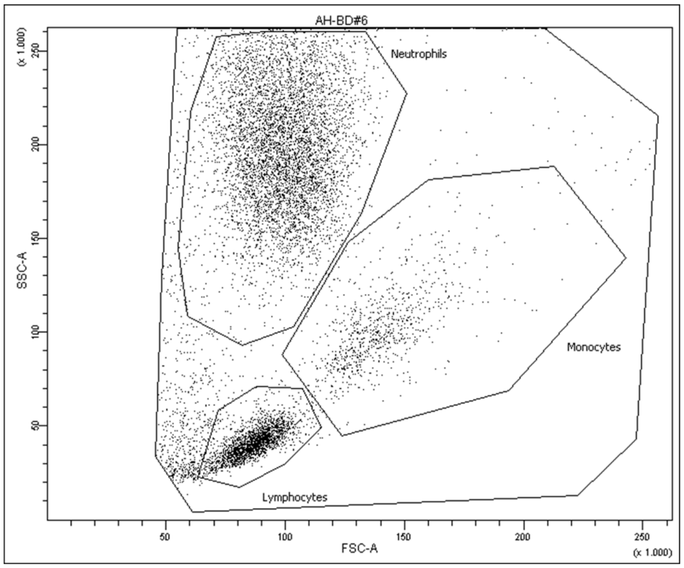

Figure S1. **Gating strategy for the identification of the immune cell subsets.**

Lymphocytes, monocytes and granulocytes (neutrophils) were identified by flow cytometry according to cell forward scatter (FSC) and side scatter (SSC) reflecting cell size and complexity.

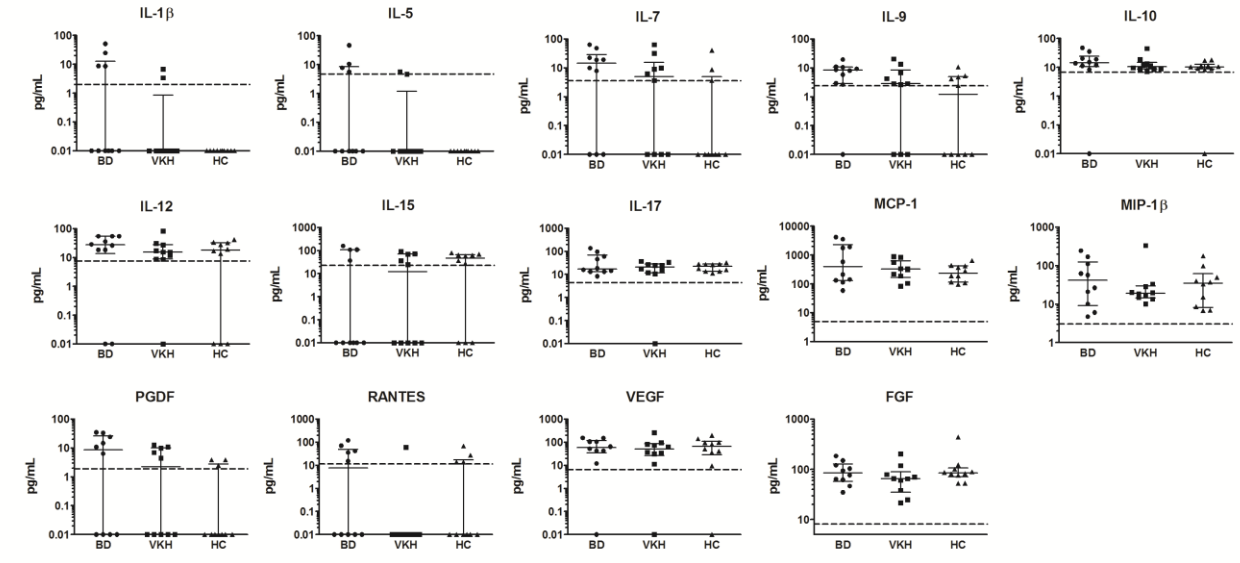

Figure S2. Distribution of the cytokines which showed a comparable expression in AH between BD, VKH and HC.

Dot plot visualization of cytokine concentrations (pg/ml) in AH from patients with BD (n=10), VKH (n=10) and HC (n=10). Horizontal lines show the median ± IQR. Dotted lines indicate the lower limits of cytokine detection. Data were analyzed by Kruskal-Wallis test with Dunn's correction for multiple comparisons.

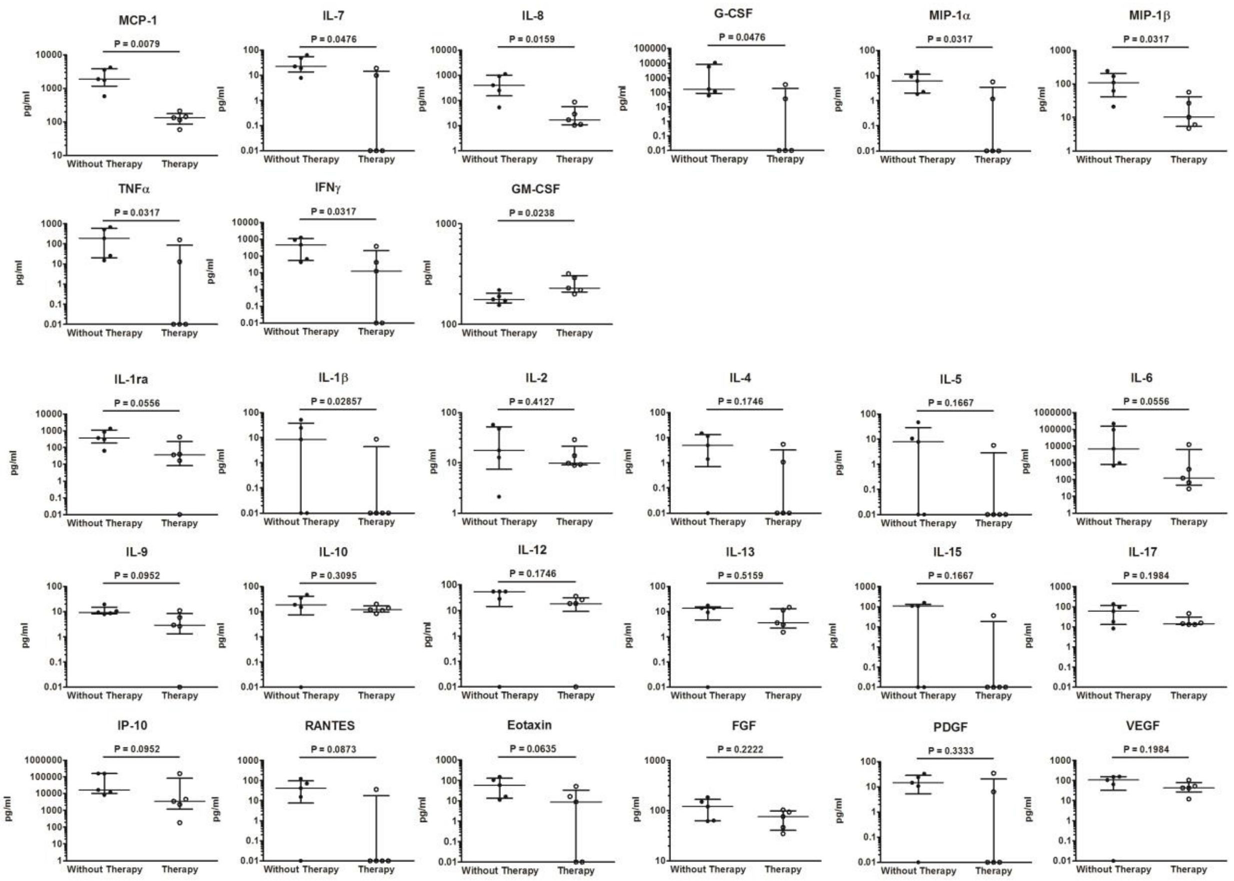

Figure S3. Cytokine concentrations in BD patients with and without therapies at the moment of AH collection.

Dot plot visualization of cytokine concentrations (pg/ml) in AH from patients with BD under therapies (n=5) and without therapies (n=5). Horizontal lines show the median ± IQR. P values obtained with Mann-Whitney test are shown. Following Bonferroni correction for multiple comparisons statistical significances are lost.

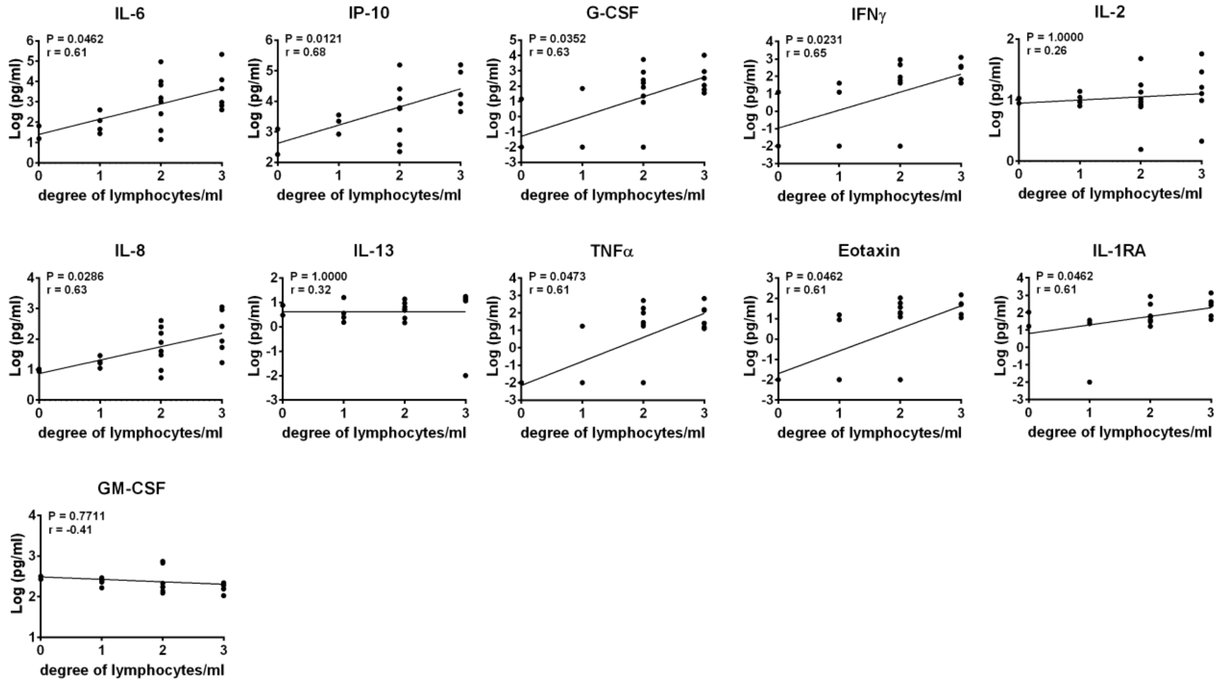

Figure S4. Correlation of cytokine concentrations with the degree of lymphocytes in AH.

Correlations between cytokine concentrations and the semi-quantitative degrees of lymphocytes/ml in AH samples are depicted (n = 20). Spearman correlation was determined followed by Bonferroni correction for multiple testing.

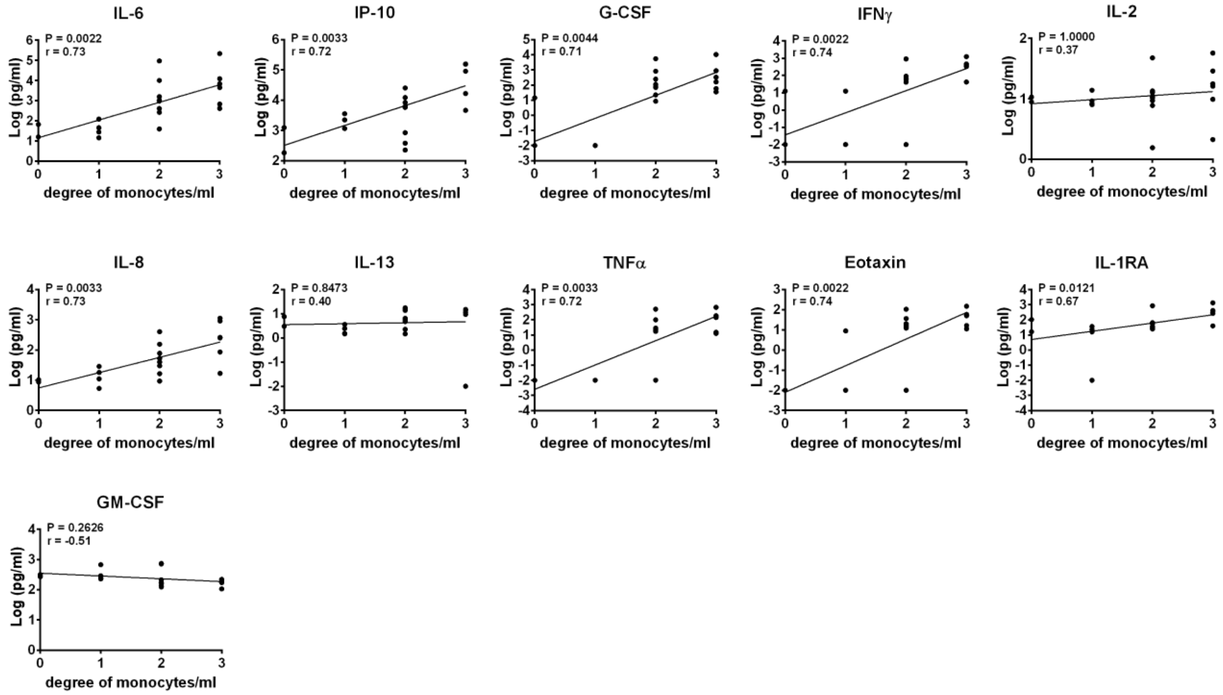

Figure S5. Correlation of cytokine concentrations with the degree of monocytes in AH.

Correlations between cytokine concentrations and the semi-quantitative degrees of monocytes/ml in AH samples are depicted (n = 20). Spearman correlation was determined followed by Bonferroni correction for multiple testing.

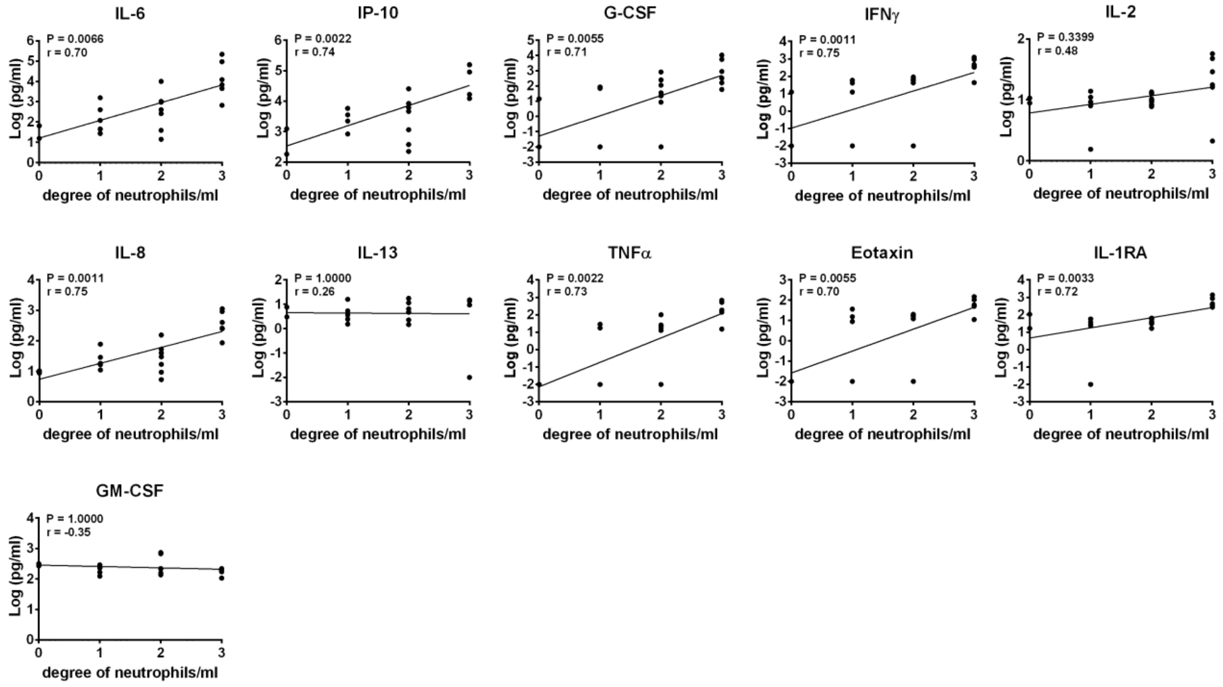

Figure S6. Correlation of cytokine concentrations with the degree of neutrophils in AH.

Correlations between cytokine concentrations and the semi-quantitative degrees of neutrophils/ml in AH samples are depicted (n = 20). Spearman correlation was determined followed by Bonferroni correction for multiple testing.

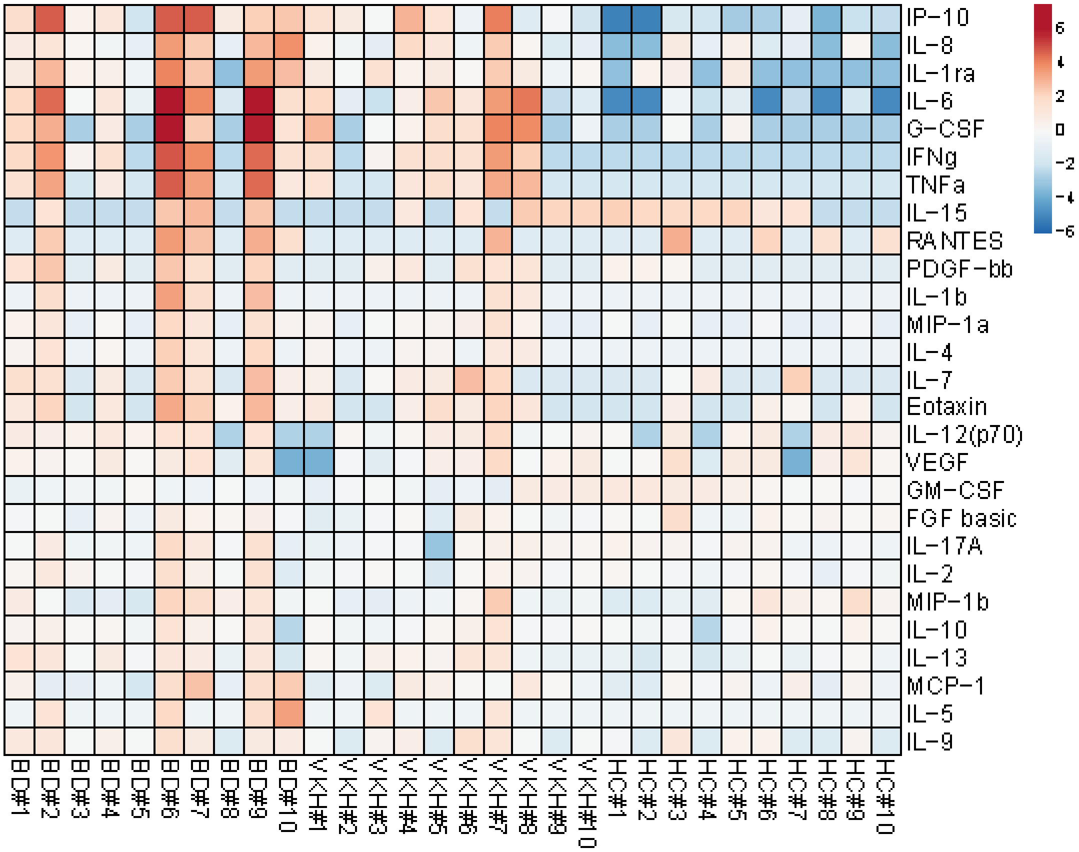

Figure S7. Heatmap of cytokine concentrations in AH from patients clustered according the clinical diagnosis and from healthy controls.

Heatmap was drawn using ClustVis software.
